# The interaction of intestinal microbiota and innate lymphoid cells in health and disease throughout life

**DOI:** 10.1111/imm.13138

**Published:** 2019-11-27

**Authors:** Stephanie C. Ganal‐Vonarburg, Claudia U. Duerr

**Affiliations:** ^1^ Department for BioMedical Research (DBMR) Bern University Hospital Universitätsklinik für Viszerale Chirurgie und Medizin Inselspital University of Bern Bern Switzerland; ^2^ Institute of Microbiology, Infectious Diseases and Immunology Charité – Universitätsmedizin Berlin Germany

**Keywords:** early life, infectious diseases, innate lymphoid cells, microbiota

## Abstract

Immunity is shaped by commensal microbiota. From early life onwards, microbes colonize mucosal surfaces of the body and thereby trigger the establishment of immune homeostasis and defense mechanisms. Recent evidence reveals that the family of innate lymphoid cells (ILCs), which are mainly located in mucosal tissues, are essential in the maintenance of barrier functions as well as in the initiation of an appropriate immune response upon pathogenic infection. In this review, we summarize recent insights on the functional interaction of microbiota and ILCs at steady‐state and throughout life. Furthermore, we will discuss the interplay of ILCs and the microbiota in mucosal infections focusing on intestinal immunity.

## Graphical abstract

Immunity is shaped by commensal microbiota. Recent insights will be discussed on the functional interaction of microbiota and innate lymphoid cells at steady‐state and throughout life in health and disease.

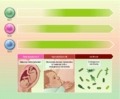

**Abbreviations**
AhR: arylhydrocarbon receptor
AOM: azoxymethane
CCR6: CC chemokine receptor 6
CD: cluster of differentiation
CHILP: common helper ILC precursor
CLP: common lymphoid progenitor
DC: dendritic cell
ES: excretory secreted
FRC: fibroblastic reticular cells
GF: germ‐free
GM‐CSF: granulocyte monocyte colony‐stimulating factor
HAART: highly active antiretroviral therapy
HpARI: *Heligmosomoides polygyrus* alarmin release inhibitor
IBD: inflammatory bowel disease
Id2: inhibitor of DNA‐binding 2
IFN: interferon
IgA/G: immunoglobulin A/G
IL: interleukin
ILC: innate lymphoid cell
ILF: intestinal lymphoid follicle
Lgr5: leucine‐rich repeat‐containing G‐protein‐coupled receptor 5
LTi cell: lymphoid tissue inducer cell
MHC: major histocompatibility complex
NCR: NK cell receptor
NK cell: natural killer cell
pDC: plasmacytoid dendritic cell
RA: retinoic acid
Rag: recombination‐activated gene
Reg3: regenerating islet‐derived protein 3
RORyt: retinoic acid‐related orphan receptor gT
SFB: segmented filamentous bacteria
SLO: secondary lymphoid organs
SPF: specific pathogen‐free
Tfh: T follicular helper cell
Th: T helper
TLR: Toll‐like receptor
TNF: tumour necrosis factor
Treg: regulatory T‐cell
TRUC: *Tbx21* *^−^* ^/−^ *Rag2* *^−^* ^/−^ ulcerative colitis
TSLP: thymic stromal lymphopoietin
WT: wild‐type

## Introduction

The role of the immune system is to protect our body from a variety of foreign and potentially harmful substances, which we are exposed to throughout life. This is especially true at the inner and outer body surfaces, such as the airways, the skin, the intestine and the urogenital tract, where the body constantly encounters non‐self‐antigens present in the food or the environment, potential pathogens, and microbes naturally colonizing these surfaces. The latter consist of bacteria, viruses, archaea and fungi, collectively referred to as commensal microbiota.

Colonization with commensal microbiota starts at birth, when the newborn leaves the sterile environment of the womb and gets in contact with the first colonizing bacteria in the birth canal. Throughout the first 1–2 years of life, the human microbiota is rather unstable and subject to change, before it reaches an astonishingly stable composition that stays throughout adulthood if external factors, such as diet, hygiene and health state are unaltered. It is only in the elderly, that the composition of the human microbiota commences to change again.^1^, ^2^ Commensal microbiota and the host live in a mutualistic relationship. While the microbiota contributes to digestive functions of the host, produces important vitamins, for example vitamin K and B group vitamins, and protects the body from invasion with pathogens, it also importantly matures the host immune system, both at mucosal and systemic sites (for reviews, see Refs^3^, ^4^). The presence of such high numbers of bacteria in and on the mammalian body is also potentially harmful, as they might induce inflammatory responses. Tight barriers and regulatory mechanisms need to be in place in order to prevent invasion of the microbes into the body and inappropriate inflammatory reactions, respectively.

Components of the innate immune system are predominantly present at mucosal sites and help maintain the host−commensal mutualism. Besides mechanical barriers, such as tight junctions between the intestinal epithelial cells and mucus produced by goblet cells, haematopoietic cells, such as mononuclear cells and innate lymphoid cells (ILCs), are important effector cells of the innate immune system.^3^ ILCs derive from the common lymphoid progenitor in the bone marrow and are by definition lymphocytes. They are predominantly found at mucosal sites, such as the intestine and the airways, react fast during an immune response, and lack antigen‐specific receptors – all typical features of innate immune cells.^5^ ILCs can be classified into five subsets, type 1 ILCs (ILC1s), type 2 ILCs (ILC2s), type 3 ILCs (ILC3s), lymphoid tissue inducer cells (LTi), which are often classified as a subset of ILC3s, and natural killer (NK) cells.^6^ ILC1s express the transcription factor T‐bet and produce interferon (IFN)‐γ and tumour necrosis factor (TNF)‐α. Similarly, NK cells also express T‐bet and produce IFN‐γ and TNF‐α. In addition, NK cells can lyse target cells in a cell‐contact‐dependent manner through the release of perforin and granzymes. Classical NK cells express the transcription factor Eomes and are developmentally distinct from ILC1s. Only a minor subset of Eomes‐positive, granzyme and perforin‐expressing ILC1‐like cells has been described to be involved in tumour surveillance in mice.^7^ In addition, human intraepithelial ILC1s express Eomes.^8^, ^9^ ILC2s are dependent on the expression of GATA‐3 and release mainly IL‐5 and IL‐13, thus contributing to T helper (Th)2‐responses. Both ILC3s and LTi cells are characterized by the expression of the transcription factor retinoic acid‐related orphan receptor gT (RORγt). While ILC3s produce IL‐22, IL17A and IL17F, LTi cells are mainly involved in lymphoid organ formation through the production of lymphotoxins.^6^, ^10^, ^11^ Recently, a regulatory ILC population, which also develops from the common lymphoid progenitor (CLP) in an Id3‐dependent way, was described to be the main source of immunomodulatory IL‐10 in the intestine following inflammation.^12^

Numerous functions of ILCs during homeostasis, immune responses and diseases have been described, and more are to be revealed. Interestingly, many of these either involve signals from the commensal microbiota or contribute to host−microbial mutualism. In the following, we aimed to summarize identified interactions between ILCs and the microbiota during health and disease throughout life.

## ILC development and microbiota in prenatal and early life

Although adult ILCs derive from the CLP in the bone marrow giving rise to the inhibitor of DNA‐binding 2 (Id2)^+^ common helper ILC precursor (CHILP), which finally differentiates into all ILC subsets expect NK cells,^13^ certain fetal and perinatal ILCs can derive from a progenitor found in the fetal liver. RORyt‐expressing LTi cells were first identified at embryonic day E13·5–E15·5 in the lymph node and Peyer’s patch anlagen to be responsible for the induction of these secondary lymphoid organs (SLO).^14^, ^15^, ^16^, ^17^, ^18^ The formation of these early LTi cells seems to be independent of the presence of the commensal microbiota as germ‐free (GF) mice harbour normal numbers of lymph nodes and Peyer’s patches.^16^, ^19^ In contrast, maternal dietary components, such as retinoic acid (RA), were shown to be crucial to maintain this LTi cell population during fetal development and consequently for the correct induction of SLO.^20^ Interestingly, microbiota is directly regulating the availability of RA.^21^ Specifically, *Clostridia *species influence epithelial RA synthesis.^22^ A similar population of RORyt^+^ ILC3s is required to induce cryptopatches and intestinal lymphoid follicles (ILFs) after birth.^23^, ^24^, ^25^, ^26^ Lin^−^ IL‐7Ra^+^ Flt3^−^ a_4_b_7_^+^ progenitors present in the fetal liver can give rise to ILC3s that can be found in the intestinal lamina propria at the time of birth.^27^, ^28^ The population of RORyt‐expressing ILCs in the lamina propria shortly after birth consists of mainly CCR6^+^ c‐kit^hi^ LTi cells, which seed the intestinal lamina propria during fetal development, and low numbers of CCR6^−^c‐kit^lo^ ILC3s that expand strongly within the first 2–4 weeks of birth and that can acquire NK cell markers.^29^, ^30^ The expansion of the latter was shown to be dependent on signals originating from the maternal microbiota as shown in a model of reversible gestational colonization during pregnancy.^31^ Arylhydrocarbon receptor (AhR) ligands produced by the maternal microbiota were transferred to the offspring postnatally through the milk and permanently increased the absolute number of NKp46^+^ ILC3s in the offspring small intestinal lamina propria starting at postnatal day 14. This is in line with three studies comparing adult specific pathogen‐free (SPF) and GF mice, which  reported a reduced number of intestinal lamina propria NCR^+^ ILC3s in GF mice.^32^, ^33^, ^34^ This area is, however, subject to controversy as other studies showed a negative influence of missing microbiota on the different ILC3 subsets.^35^ In a separate study using *RORc* ^Cre^ × *AhR* ^fl/fl^ mice and experimentally purified AhR‐ligand‐enriched or depleted diets, it was demonstrated that CCR6^−^ ILC3s were strongly reduced in numbers in the absence of any Ahr signalling, which resulted in the absence of intestinal cryptopatches and ILFs.^36^ Natural ligands of the AhR include agonists derived from cruciferous vegetables in the diet as well as indoles produced by members of the microbiota. Although, this study did not investigate the influence of microbiota‐derived Ahr ligands on the proliferation of CCR6^−^ ILC3s, it is likely to speculate that AhR ligands derived from not only the maternal microbiota but also from the endogenous microbiota of the offspring contribute to the homeostasis of this ILC population. This is supported by the observation that GF mice show a delay in the maturation of cryptopatches into ILFs.^37^ A high degree of plasticity has been described for intestinal ILC3s. While the T‐bet induced upregulation of NK cell receptors (NCRs) on ILC3s was dependent on the presence of the commensal microbiota as described above,^13^, ^32^, ^34^ the loss of RORyt‐expression in NCR^+^ ILC3s and their switch to a more ILC1‐like phenotype was prevented in colonized compared with GF mice.^33^

A recent publication showed that perinatal signals leading to expansion of neonatal ILC2s are important to maintain even the adult pool of ILC2s.^38^ This perinatal expansion of ILC2s was independent of commensal microbiota as it occurred also in GF mice. As interactions between an individual SPF consortium and a defined diet are very complex, it is not unlikely that a combination of dietary and microbial signals contributes to this event. It is also known that ILC2 numbers in the intestine of GF adult mice are higher compared with those in colonized animals, reflecting the general shift towards Th2‐type immune responses in GF mice.^39^ In contrast to the ILC3s, transient maternal microbial exposure during gestation did not change the ILC2 numbers in the offspring intestine.^31^

The role of microbiota in the differentiation of ILC1s and NK cells during early life has not been investigated in detail. However, cell numbers in the different lymphoid organs were unaffected by the commensal microbiota both in young and adult mice wherever investigated.^40^ A study on the human ILC1 subset by Spits and colleagues has suggested that colonization of the neonatal intestine is triggered through microbiota colonization after birth as this subset seems absent in the fetal intestine. In addition, they show that this ILC1 subset has the potential to differentiate into RORγt‐expressing ILC3 during adulthood in the presence of IL‐23 and RA, the latter being partially regulated by the commensal microbiota.^41^

**Figure 1:**
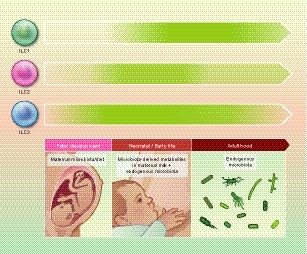
Influence of the commensal microbiota on innate lymphoid cells (ILCs) throughout life. Although mammals get colonized with commensal microbiota at the time of birth, microbial signals originating from the maternal microbiota can reach the offspring already during the fetal stage via the placenta and during neonatal life through maternal milk. In addition, both ILC3s and ILC2s have been described in the fetal liver. Microbial influences on ILC development, maintenance and function are thus possible throughout life. The current knowledge on microbial influences on ILC numbers and functions was used to estimate the importance of commensal microbiota‐derived signals on intestinal ILCs at the different stages of life (fetal, neonatal, adult). The strength/importance of the microbial influence is indicated here by the shade of green colour used in the arrows. ILC3s seem to be the ILC population that is most strongly dependent on microbial signals, and these are especially important in establishing the intestinal ILC3 pool during early life through signals originating also from the maternal microbiota during pregnancy. ILC2s are generally slightly less dependent on the presence of commensal microbiota, especially in early life. However, a slight increase in cell numbers has been described in adult germ‐free (GF) mice. ILC1s are believed to be absent during fetal life, and the influence of microbiota appears to be stronger the more mature and possibly pro‐inflammatory the intestinal microbiota has become.

A study by Gury‐BenAri *et al*. has characterized 15 different existing intestinal ILC subsets at single cell level in regard to transcriptomic activity and chromatin landscape in SPF, GF and antibiotic‐treated mice. While antibiotic‐treated and GF mice exhibited ILC subsets with very similar expression profiles, colonized animals clustered separately in most subsets. ILC1 and ILC2 subsets were most affected by the absence of commensal microbiota and acquired a phenotype that more closely resembled ILC3 subsets.^42^ In conclusion, while ILC3s seem to be mainly influenced by microbial signals during early life, ILC2s and ILC1s are more shaped through microbiota during adulthood (Fig. 1). 

## Microbiota and ILC interactions during steady‐state in adulthood

### Contribution of microbiota to the establishment of a physical barrier: epithelial integrity

The different ILC subsets are predominantly found at mucosal sites, and the intestinal lamina propria is home to all three main subsets of ILCs: ILC1s, ILC2s and ILC3s. One of the first discovered functions of ILCs is their role in maintaining a tight and healthy intestinal barrier, and thus contributing to control the intestinal microbiota. ILC3 produce the cytokine IL‐22, which can act on the IL‐22 receptor expressed exclusively on non‐haematopoietic cells in the gut, mainly intestinal epithelial cells.^43^ IL‐22 predominantly acts on Paneth cells to induce the release of the antimicrobial proteins Reg3b, Reg3y, S100A8, S100A9.^44^ IL‐22 produced by ILCs was also shown to enhance proliferation of Lgr5^+^ intestinal epithelial stem cells in murine and human organoids and in an *in vivo* mouse model.^45^ Recently, Gronke *et al*. demonstrated that ILC3‐derived IL‐22 induces the DNA damage response in intestinal epithelial cells, protecting the epithelial layer from exogenous genotoxic stress, thus limiting tumorigenesis.^46^ The production of IL‐22 by both CCR6^−^ and CCR6^+^ ILC3s is stimulated by commensal microbiota as shown by the use of GF and antibiotic‐treated mice.^29^ ILC3‐derived IL‐22 induces the expression of various other factors in the epithelium, including tight and gap junction proteins, mucins, and cytokine receptors that contribute to the homeostasis and barrier function of the epithelium (for review, see Ref.^47^). Two reports illustrated that ILC3s are crucial in preventing dissemination of intestinal commensals to peripheral tissues: the titres of serum IgG specific for intestinal commensal bacteria were elevated in RORyt‐deficient mice,^48^ and Rag^−/−^ mice exhibited systemic translocation of Alcaligenes species and systemic inflammation upon depletion of ILCs using anti‐Thy1 antibodies.^49^

Epithelial‐derived cytokines, such as IL‐25, IL‐33 and thymic stromal lymphopoietin (TSLP), are regulated by commensal microbiota and act on ILC2 maintenance and function.^35^, ^50^ IL‐13 produced by ILC2s can act on intestinal epithelial stem cells and bias their differentiation into goblet cells and tuft cells, promoting mucus production by goblet cells and environmental sensing capacities of the intestinal epithelium through tuft cells. A positive feedback loop exists between ILC2s and Tuft cells as ILC2‐derived IL‐13 induces further IL‐25 secretion by Tuft cells.^51^ In addition, IL‐13 secreted by ILC2s supports self‐renewal of intestinal epithelial stem cells.^52^

IFN‐γ and TNF‐α, which are also produced by T‐bet^+^ ILC1s, are known to increase permeability of the intestinal epithelial cell barrier as shown in bacterial translocation studies using monolayers of human intestinal epithelial cells.^53^, ^54^ The absence of T‐bet in the innate immune system also directly affects microbiota composition as presented further down.

As outlined above, one main function of ILCs lies within the strengthening of epithelial barriers at mucosal surfaces through various mechanisms and thus regulating the translocation of microbial members into the body. This is supported by the location of most ILCs within mucosal tissues and the resulting proximity to such epithelial barriers.

### Microbiota, ILCs and other members of the innate immune system

Innate lymphoid cells (ILCs) carry several cytokine receptors and can thus react to cytokines released by other innate immune cells. Intestinal mononuclear phagocytes produce IL‐23, IL‐1α and IL‐1β in a microbiota‐dependent manner, which induce IL‐22 and IL‐17 production by ILC3s.^55^, ^56^, ^57^, ^58^ These cytokines are mainly released by CX3CR1^+^ macrophages and CD103^+^ CD11b^+^ dendritic cells (DCs) resident in the intestinal lamina propria.^57^, ^59^, ^60^ Microbiota‐induced IL‐1β also induces the release of granulocyte monocyte colony‐stimulating factor (GM‐CSF) by ILC3, which in turn promotes mononuclear phagocytes to produce regulatory components, such as IL‐10 and RA.^57^ The latter are important to promote regulatory T‐cell (Treg) differentiation and expansion in the intestine to ensure intestinal homeostasis.

Splenic NK cells in GF and antibiotic‐treated mice are largely unresponsive when stimulated to produce IFN‐γ or exert specific lysis of target cells following Toll‐like receptor (TLR) ligand exposure *in vivo*. This was due to functionally impaired DCs in the absence of microbiota that were unable to produce the NK priming cytokines IFN‐I and IL‐15.^40^

### Microbiota, ILCs and adaptive immune responses

While ILC function is influenced by commensal microbiota, ILCs themselves can influence adaptive immune cells, which in return help keeping the host−microbial mutualism in check. The largest set of data is available on the role of ILC3s in regulating T‐ and B‐cell responses. The CCR6^+^ subset of ILC3s present in the colonic lamina propria and mesenteric lymph nodes expresses major histocompatibility complex (MHC)‐II and can act as antigen‐presenting cells to negatively select commensal bacteria‐specific CD4^+^ T‐cells. The elimination of microbiota‐specific T‐cells was very important to prevent low‐grade systemic and spontaneous intestinal inflammation.^61^, ^62^ An independent study demonstrated that splenic but not intestinal MHC‐II‐expressing ILC3s can be activated through microbiota‐derived IL‐1β to express co‐stimulator molecules and thus drive CD4^+^ T‐cell and B‐cell responses *in vivo*.^63^ Similarly, microbiota‐derived signals during colitis induced the expression of the co‐stimulatory molecule OX40 on MHC‐II‐expressing ILC3s, which could subsequently activate intestinal T‐cells.^64^ ILC3s were also shown to localize within the MLN to the interface between the T‐ and B‐cell zone to regulate T follicular helper cell (Tfh)‐dependent germinal centre reactions.^65^ This was dependent on MHC‐II expression and antigen presentation by ILC3s and significantly reduced colonic IgA^+^ B‐cell responses to commensal microbiota, thereby contributing to host−commensal mutualism.^66^ At the same time, lymphotoxin alpha expression by ILC3s was critical to control T‐cell homing to the gut, representing another influence of ILC3s on T‐dependent IgA production.^67^ However, ILC3s not only affect T‐dependent, but also T‐independent IgA induction via the production of BAFF and APRIL in the spleen,^68^ and through expression of the lymphotoxin beta receptor and interaction with DCs in intestinal lymphoid follicles.^69^

ILC2s can likewise influence CD4^+^ T‐cell responses. ILC2s were shown to skew CD4^+^ T‐cell differentiation and activation towards Th2 instead of Th1 in a cell−cell contact dependent manner.^70^, ^71^, ^72^ A subset of ILC2s also express MHC‐II and can present antigens to CD4^+^ T‐cells.^70^, ^71^ ILC2s could potentially also affect the generation of intestinal IgA, and therefore influence commensal microbiota, through the release of IL‐5, which is favourable for IgA induction in B cells, and through IL‐6 that promotes B‐1 cell proliferation.^73^

### Impact of ILCs on microbiota composition

Knowing that ILCs have direct effects on intestinal barrier integrity on several levels, including the regulation of intestinal epithelial cells, of innate immune populations and of adaptive immune responses, it is expected that the absence of one or several ILC populations goes along with an altered host−microbial mutualism and ultimately with microbial dysbiosis. Still, only few studies are available that have addressed commensal microbiota composition in mice deficient in ILC populations or with altered ILC function. Such studies are also challenging as cage effects are prone to affect the results, and the requirement for littermates can be a limiting factor and is often neglected.^74^, ^75^ While microbiota diversity and phylum composition were unaltered in the absence of ILC3s (*RORc* ^Cre^ × *Id2* ^fl/fl^), these mice exhibited higher levels of segmented filamentous bacteria (SFB) as well as *Clostridiales* species.^76^ Mice lacking the AhR in ILC3s, which goes along with reduced numbers of ILC3s, or lymphotoxin alpha in ILC3s, also carried more SFB,^67^, ^77^ corroborating the hypothesis that SFB, which are associated with a Th‐17‐mediated inflammatory phenotype,^78^ are under control of ILC3s. Several studies addressed microbiota composition in models of IL‐22 deficiencies. IL‐22‐deficient mice harboured a dysbiotic colonic microbiota with colitogenic potential compared with wild‐type (WT) control mice, which was transmissible to WT animals if adult animals of the two strains were co‐housed.^79^ Unfortunately, no littermates were addressed to understand the role of IL‐22 in protecting from the acquisition of a colitogenic microbiota in early life as it has been shown for the presence of TLR5 in the neonatal period.^80^ Another study demonstrated that Id2 expression in ILC3s was important for the generation of IL‐22, which maintained a healthy microbiota that exhibited early colonization resistance to *Citrobacter rodentium*.^76^ ILC3s in the skin were recently shown to control the growth of the sebaceous glands and consequently the release of antimicrobial lipids, enabling the establishment of a healthy skin microbiota.^81^

T‐bet depletion in *Rag* ^−/−^ mice exhibited commensal dysbiosis associated with susceptibility to colitis in one but not another mouse colony.^82^, ^83^ Since a subset of ILC3s, ILC1s, and NK cells express or depend on the transcription factor T‐bet, it is difficult to know which ILC subset is responsible for this phenotype. T‐bet depletion in the innate immune system in the colony harbouring the colitic microbiota went along with stronger IL‐17 expression by the remaining ILC3s.^83^ In a later study, if T‐bet was depleted ubiquitously (*Tbx21* ^−/−^) or in NCR^+^ ILCs (*Ncr1* ^iCreTg^ × *Tbx21* ^fl/fl^) in either immunocompetent or compromised mice, ILC2 frequency and function in the colonic lamina propria, spleen and lymph nodes were increased, promoting protective mucosal immunity during worm infections.^84^ This was mediated through increased IL7Rα expression by ILC2s in the absence of T‐bet. To our knowledge, intestinal microbiota composition in ILC2‐deficient mice has not yet been investigated.

## Microbiota – ILC axis during infectious diseases

### Microbiota and ILCs in parasitic infections

#### Helminth infections

Approximately one‐third of the world’s population is infected with helminths.^85^ Helminth infections greatly influence the host’s immune response and dampen immunity to co‐infections.^86^ Type 2 signature cytokines are induced triggering goblet cell differentiation, mucin release and smooth muscle contraction commonly referred to as ‘weep and sweep’ mechanism to expel helminths. Not surprisingly, helminth infections disturb the microbial composition of the host. Depending on the infecting helminth, different shifts of the microbiota towards specific bacterial species have been reported with an imbalance of *Lactobacilli *species in C57BL/6 laboratory mice.^87^, ^88^ Helminths are also able to act directly on the host’s immune response by the release of excretory secreted (ES) vesicles carrying several different immunomodulators.^89^ Upon helminth infections, intestinal ILC2s are activated mainly by alarmins such as IL‐25 and IL‐33.^90^, ^91^ Indeed, ILC2s have been firstly identified in detail upon infection with the intestinal helminth *Nippostrongylus brasiliensis *(*N. brasiliensis*).^73^, ^92^, ^93^ Due to their fast release of type 2 cytokines, such as IL‐13, ILC2s accelerate worm expulsion by triggering epithelial immunity. Release of tuft cell‐derived IL‐25 activates ILC2s to increase IL‐13 expression, which then again acts on the epithelium. This positive feedback loop of cooperate action of ILC2s and epithelial cells is key to expel and eliminate the pathogen.^51^, ^94^, ^95^ However, helminth‐derived *Heligmosomoides polygyrus* alarmin release inhibitor (HpARI), which is able to neutralize ILC2 activating IL‐33, dampens protective type 2 immunity.^96^ Whether ILC populations and specifically ILC2s are able to directly sense and react to helminth‐derived ES vesicles will be of great interest for future studies. Helminth infections can trigger malnutrition and worsen disorders including vitamin A deficiency. The vitamin A metabolite RA is essential for the intestinal immune response upon infection: decreased ILC3 levels but increased number and activity of ILC2s, such as increased IL‐13 secretion, have been reported in helminth infections (*Trichuris muris*;* T. muris*) in vitamin A‐insufficient mice.^97^ The metabolism of ILC2 relies highly on fatty acid metabolism at steady‐state as well as upon *T. muris* infection on RA‐triggered malnutrition.^98^ AhR‐deficient ILC2s show enhanced activity and thereby acceleration of clearance of helminths (*Heligmosomoides polygyrus bakeri*).^99^ This is due to decreased recruitment of the transcriptional determinant Gfi1 to the *Il1rl1* locus in genetically induced AhR‐deleted ILC2s.

#### Toxoplasma gondii

**Figure 2:**
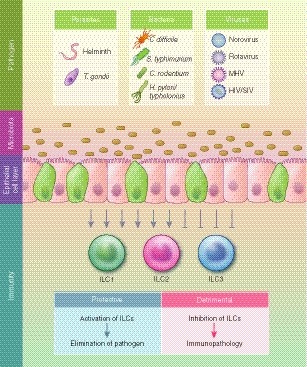
Intestinal infections lead to perturbations of the microbiota and alter innate lymphoid cell (ILC) activity. Parasitic, bacterial and viral infections influence microbiota composition and function as well as the activity of ILCs. Depending on microbial components and immunomodulators induced by pathogens, the ILC activation can be protective or detrimental, resulting in either pathogen elimination or immunopathology, respectively.

The intracellular parasite *Toxoplasma gondii *(*T. gondii*) is mainly transmitted in the cyst stage, and induces dysbiosis and a shift of the microbiota to Enterobacteriaceae.^100^ A strong type 1 immune response is generated by IFN‐γ producing innate and adaptive lymphoid cells. Indeed, T‐bet‐dependent ILC1s are able to control *T. gondii* infections by their release of IFN‐γ and TNF‐α.^13^ An additional T‐bet‐dependent population of intraepithelial lymphocytes with an ILC1 profile has been reported recently.^101^ These NKp46^−^ CD8αα^−^ Ly49E^+^ IELs express IFN‐γ upon *T. gondii* infection, and thereby promote the type 1 immune response to eliminate *T. gondii*. A recent report identified plasticity of NK cells to ILC1s using Eomes‐fate‐map mice in *T. gondii* infection highlighting how closely related these populations are.^102^ Moreover, not only parasitic but also bacterial and viral infections impact on microbiota composition and ILCs functionality (Fig. 2), which will be discussed in the next paragraphs.

### Microbiota and ILCs in bacterial infections

#### Gram‐positive bacteria – *Clostridium difficile* infections

Microbiota is severely reduced and colonization resistance lost upon broad‐spectrum antibiotics treatment, which increases the susceptibility to infection by the Gram‐positive bacterium *Clostridium difficile* (*C. difficile*). In North America, *C.* *difficile* infects several hundred thousand people every year, and represents an important health threat especially for immune‐compromised and hospitalized patients. Adaptive immune responses and innate immunity cooperate to eliminate *C. difficile*, which establishes its infection by the toxin‐controlled inhibition of epithelial GTPases thereby destroying the epithelial barrier.^103^ Innate lymphoid populations are important in the defense against *C. difficile* reported by studies in ILC‐deficient mice.^104^, ^105^ Transfer experiments of ILCs revealed that especially ILC1s and ILC3s contribute through the secretion of IFN‐γ and IL‐22 in the acute phase of *C. difficile* infection.^104^ In a recent report, an additional mechanism based on IL‐33 and its induction of ILC2s in *C. difficile* infection was described: upregulation of IL‐33 during infection induces ILC2s thereby acting as a protective immune mechanism. Furthermore, in human fecal transplant patients, the transfer of microbiota induced IL‐33 and thereby triggered a protective immune response.^106^ These reports indicate that all helper ILC populations are involved in resolving *C. difficile* infections; however, their importance may be dependent on the phase of the infection. As mentioned earlier, *C. difficile* infections are successfully treated by the therapeutic approach of fecal transplants to restore microbiota and eliminate the ecological niche for *C. difficile.* 107 Whereas this therapeutic approach has had great success, a current trial has led to the death of one patient by transferring microbiota including multi‐resistant bacteria,^108^ underlining the need for accurate and detailed screening of potential pathogens in transplants. Conversely to the immune response in *C. difficile* infections, it is still unknown whether and to which extent ILCs contribute to the short‐ and long‐term changes upon fecal transplant in humans.^109^ Moreover, susceptibility to *C. difficile* increases with age; however, direct links to microbiota dysbiosis and/or ILC populations have not yet been reported in these conditions.

#### Gram‐negative bacteria – Salmonella, Citrobacter and Helicobacter infections

Non‐typhoidal *Salmonella* (Gram‐negative) species such as *Salmonella typhimurium* are transmitted by contaminated food. Intestinal infections occur fast, accompanied by stomach pain and diarrhoea, and are usually cleared within several days in healthy individuals. Direct links to ILCs during *Salmonella* infections in humans have not been reported, but it has been suggested that ILC1s play a role based on the observation that ILC1s are not present in the fetal human gut in contrast to ILC2s and ILC3s, and thus may be triggered by commensal bacteria acquired upon birth, which include Gram‐negative bacteria.^110^, ^111^ In mice, *S. typhimurium* is only able to establish an intestinal infection upon loss of the host’s colonization resistance experimentally induced by a one‐time treatment with the antibiotic streptomycin.^112^ Upon *Salmonella* infection, adaptive and innate immunity including epithelial cells and ILCs work in concert to eliminate the bacteria. IFN‐y is key to fight the infection, and its expression has been reported to be shared by conventional NK cells as well as ILC3s during *Salmonella* infection in mice with T‐bet^+ ^CCR6^− ^RORγt^+^ ILC3s being the strongest IFN‐y producers.^29^ In addition, intestinal IL‐22 expression triggered by intestinal epithelial cell‐derived RA affects antimicrobial peptide expression and bacterial load in *S. typhimurium* infection.^22^

Enterohaemorrhagic* Escherichia coli* (EHEC) infections, which are often detrimental in humans, are modelled by infection with the Gram‐negative bacterium *C. rodentium* in mice. ILCs are essential in the defense against *C. rodentium* on several levels. ILC‐derived IL‐22 acts on non‐haematopoietic cells to initiate an innate immune defense program against *C. rodentium.* 113, 114 CD4^+^ LTi cells have been identified as an important source of IL‐22 upon *C. rodentium* infection in mice^115^ as well as ILC3s, specifically, CCR6^low/−^ ILC3s. Interestingly, the latter population is dependent on ligand‐induced stimulation of AhR, as discussed previously. Whereas ILC3s are supported by AhR stimulation and needed to fight off *C. rodentium* infection, ILC2s are inhibited by AhR signalling in helminth infections.^99^ Retinoic acid is essential for ILC3 activity during *C. rodentium* infection. Vitamin A‐insufficient mice are more susceptible to *C. rodentium* due to a reduction in IL‐22‐expressing ILC3s.^97^

Antigen presentation by ILC3s via MHC‐II is important to maintain the mucosal barrier at steady‐state, but also upon infection. In mice lacking MHC‐II in ILC3s, infection with *C. rodentium* resulted in an increased Tfh response, elevated levels of IgA and *C. rodentium*‐specific IgA underlining that LTi‐like ILC3s are key in triggering the adaptive immune response via regulation of IgA and pathogen infections.^66^

In humans, the Gram‐negative bacteria *Helicobacter pylori* colonizes the stomach and intestine, but can also trigger colon cancer when becoming pathogenic. Li *et al*. reported an increase in Lin^+^ GATA3^+^ as well as Lin^−^ GATA3^+^ ILC2‐like populations together with a more pronounced type 2 signature limiting the protective type 1 immune response in the development of cancers.^116^ In mice*, Helicobacter typhlonius* infection triggers IL‐17A expressing ILCs by synergizing DCs derived TNF‐α and IL‐23 in the *Tbx21* ^−/−^ *Rag2* ^−/−^ ulcerative colitis (TRUC) model.^83^

### Microbiota and ILCs in viral infections

#### ssRNA viruses: Norovirus, mouse hepatitis virus and influenza virus

Norovirus (family of *Caliciviridae,* ssRNA) infections are highly contagious, and typical symptoms include diarrhoea, nausea, vomiting and abdominal pain in humans. In mice, Murine Norovirus (MNV) triggers changes of epithelial innate immunity of the small intestine in mice hypomorphic for ATG16L expression.^117^, ^118^ Because ATG16L is a risk gene for Crohn’s disease in humans, this report highlights the close link of genetic predisposition and microbial exposure. Interestingly, infections of MNV in GF mice have identified an important role of virus ‘colonization’ on the host immune system. Here, MNV mimics beneficial immune modulatory function of the microbiota and influences intestinal immune responses as well as ILC numbers and function.^39^ Whether and how MNV infection impact on microbiota and thereby immunity of ILC populations in SPF colonized mice still needs to be investigated. However, upon antibiotic treatment, MNV infection or sensing of viral ssRNA by TLR7 is able to trigger antimicrobial peptide expression as well as IL‐23 release, and thus tightening of the epithelial barrier together with increased ILC3 function in the defense against vancomycin‐resistant *Enterococcus faecium* (VRE).^119^ Interestingly, treatment of psoriasis with the TLR7‐activating drug imiquimod has been linked to colonic dysbiosis and thus to increased susceptibility to DSS colitis.^120^ However, this was not ILC3 dependent in contrast to reports from the previously discussed study.^119^

The pathogenic Mouse Hepatitis Virus (MHV; family of *Coronaviridae,* ssRNA) targets the intestine and liver, and is able to cause severe systemic disease. It is feared in animal facilities as a contamination but also used successfully to study viral pathogenicity. Here, upon oral MHV infection, fibroblastic reticular cells (FRCs) secrete IL‐15 in a MyD88‐dependent manner and thereby control ILC1s and intestinal inflammation. Dysbiosis is observed in the absence of innate immune stimulation, showing that tissue circuits between non‐haematopoietic cells, such as FRCs and haematopoietic cells including ILC1s, are key in intestinal immune responses against viruses.^121^

Although respiratory viruses, including influenza virus (ssRNA), are limited to the airways during infection and have been reported to impact systemic immunity and the intestinal microbiota.^122^, ^123^ Indeed, immunity and microbiota of the gut and lungs are closely interlinked.^124^ In humans, the microbiome of the upper respiratory tract, including nose and throat, has been shown to change upon influenza infection.^125^ Whether intestinal ILC populations are affected in influenza infection in humans has not yet been addressed. However, ILCs, specifically ILC2s, are increased upon infection with influenza A virus (IAV) in the respiratory tract in mice.^126^, ^127^, ^128^ IAV‐induced ILC2s can be detrimental for the host by inducing excessive type 2 immune responses and airway hyperreactivity independently of the adaptive immune system. Control of ILC2s by IFN is thus an important mechanism to restrain ILC2s upon IAV infection.^127^ However, ILC2s can also support wound healing upon infection by the secretion of AREG in the respiratory tract as well as in the gut during inflammation, and thereby be beneficial for the host.^126^, ^129^ Experimental factors might be involved in the different outcome of the ILC2 character including the use of different IAV subtypes in the studies, namely H3N1 and H1N1, or general variations in experimental procedures such as the different experimental time points analysed as previously discussed.^130^, ^131^

Antibiotic treatment influences not only the microbial composition in the gut (discussed above), but also has an important impact on ILC plasticity and function,^42^ as well as on immunity to respiratory infections, such as influenza virus.^132^ Here, reduced signalling via the inflammasome and TLRs leads to decreased activation and migration of DCs into the mediastinal lymph node. Thereby, the priming of T‐cells is reduced and the initiation of the immune response hampered. Moreover, reduced levels of virus‐specific T‐cells to influenza virus were reported by Abt and colleagues due to changes in macrophage function in antibiotic‐treated mice.^133^ Conversely, IAV infection has been reported to not be influenced by antibiotic treatment in immature mice.^134^ However, how antibiotic treatment influences pulmonary ILCs in pathogenic infections remains to be elucidated.

#### Lentiviruses: Human immunodeficiency virus and simian immunodeficiency virus

Infection by human immunodeficiency virus (HIV; family of Retroviridae, ssRNA) leads to acquired immunodeficiency syndrome (AIDS), an immunodeficiency characterized by a severe reduction of CD4 T‐cells. Highly active antiretroviral therapy (HAART) is used successfully to restrict viral load and maintain CD4 T‐cells. Studies to decipher the underlying mechanism in HIV infections include studying simian immunodeficiency virus (SIV)‐infected non‐human primates, HIV‐infected humanized mice and peripheral blood, as well as biopsies of HIV‐infected human individuals. HIV and SIV infection break down the intestinal barrier leading to disruption of the intestinal integrity and transition of intestinal antigens followed by systemic inflammation.^135^ Patients with HIV infections have been reported with perturbations of the bacterial microbiota together with expansion of the virome.^136^ Upon HIV/SIV infection, intestinal CD4 T‐cells as well as ILC populations, specifically ILC3s, are massively decreased.^137^ HIV is not able to directly infect ILCs as they lack both co‐receptors used by HIV for viral docking, namely CCR5 and CD4. However, human CD45^+^ Lin^−^ CD117^−^ CRTH2^−^ CD127^+^ CD56^−^ CD4^+^ cells can still be infected by HIV.^138^ In acute HIV infection, ILC1s, ILC2s and ILC3s are significantly decreased in peripheral blood and only early HAART is able to preserve ILCs. Late HAART therapy was not able to reconstitute ILCs levels.^139^ Decrease in ILC subsets in HIV infection is further confirmed by a recent report showing the reduction of intestinal ILC subsets upon chronic HIV‐1 infection and furthermore reporting an increase in CD94^+^ memory‐like NK cells.^140^ Indeed, intestinal ILCs in patients with HIV change their phenotypic appearance such as expression of the IL‐7Rα chain (CD127), which is needed to sense IL‐7, an important survival and proliferation factor.^141^ Moreover, upregulation of apoptotic signals has been reported in ILC3s induced by plasmacytoid dendritic cell (pDC)‐derived interferon upon HIV in humanized mice. Microbiota triggered IL‐2 production by ILC3 has just recently been shown to support Tregs.^142^ It is tempting to speculate that loss of IL‐2 producing ILC populations may have a direct effect on T‐cell homeostasis in HIV infection.

Furthermore, decreased ILC2 function and thus reduced IL‐13 production upon *ex vivo* stimulation of SIV‐infected non‐human primate samples has been reported.^143^ It is not yet known whether impaired ILC2 activity in HIV/SIV parallels the restraint of ILC2s in IAV by interferon, and thereby triggers the loss of intestinal barrier and the increased transition of microbial ligands from the gut. IFN regulatory mechanisms upon HIV infection may be triggered by HIV itself as well as by the expansion of the enteric virome, but this hypothesis still needs to be confirmed. Interestingly, virome expansion has been reported upon SIV infections.^144^

#### dsRNA viruses: Rotavirus

Rotavirus (family of *Reoviridae*, dsRNA) can cause gastroenteritis, with young children being predominantly affected. All three groups of IFNs are important in the defense against Rotavirus, but type III IFNs (IFN‐λ) are only acting on and triggering intestinal epithelial cell immunity.^145^ Moreover, synergy between IL‐22 produced by ILC3s and IFN‐γ has been identified,^146^ further highlighting the strong interaction of ILCs and epithelial cells during infection. Data are limited on the link between rotavirus infection and microbiota; however, the efficacy of a rotavirus vaccine (Rotarix: live, attenuated human rotavirus vaccine used in infants) and microbiota has been noted.^147^ To which extend this is linked to ILC3 activity has not yet been addressed, but would be of great interest.

## Microbiota − ILCs, cancer and aging

In this last paragraph of our review we want to highlight key aspects that are known about microbiota development and ILC function with age, and highlight their contributions to the development and defense against cancers – one of the most common age‐related diseases. As outlined previously, microbiota composition and function dramatically changes in aging individuals.^148^ This by itself may already alter ILC function, although one could also imagine that altered ILC activity with age^149^, ^150^ can impact the composition of the commensal microbiota through various mechanisms that we have reviewed above. Several studies have reported that both dysbiosis and altered ILC functionality can be involved in tumorigenesis and also in the anti‐tumour immune response. These aspects have recently been discussed in excellent reviews by Wagner and Koyasu,^151^ Panda and Colonna,^152^ Mattiola and Diefenbach,^153^ and Atreya *et al.* 154 Thus, we will only briefly touch on the role of ILCs in colon cancer, focusing on ILC3s, the ILC population with the strongest link to cancer so far.

Innate lymphoid cells can be both pro‐ and anti‐tumorigenic. Upon induction of intestinal inflammation by *Helicobacter hepaticus* and of cancer formation with the carcinogen azoxymethane (AOM), ILC‐derived IL‐22 acts on epithelial cells and induces cancer formation. Blocking of IL‐22 or ILCs by anti‐Thy1 antibody reduces colorectal cancer.^155^ IL‐22 can also be regulated by an endogenous mechanism, namely by the decoy IL‐22BP. Increased expression of IL‐22BP by T‐cells in inflammatory bowel disease (IBD) patients promotes cancer development.^156^, ^157^ Intestinal inflammation in IBD patients regularly triggers the development of cancer,^158^, ^159^ further confirming that inflammation is an additional hallmark of cancer.^160^ However, the protective role of IL‐22 in intestinal homeostasis can be extended to colon cancer. Recently, AhR‐induced IL‐22 in ILC3s was shown to reduce formation of epithelial stem cells in the colon upon AOM treatment. The underlying mechanism was elucidated by using the *Confetti* system enabling to trace IL‐22 receptor expressing or non‐expressing crypts. IL‐22‐deficient crypts lost the control of apoptosis through the DNA damage response. This dysregulation triggered the formation of colon cancer upon AOM treatment, highlighting the importance of IL‐22 in colon cancer carcinogenesis.^46^ In the same study, they could also demonstrate that dietary‐derived AhR ligands directly induce genotoxic stress in intestinal epithelial cells, while at the same time promoting IL‐22 production in ILC3s, which in return switches on the DNA damage response in intestinal epithelial cells. These studies further underline that ILC3s are key players in homeostatic but also in inflammatory conditions at the interface between the environment and the body. For certain, aging and cancers will be of interest in future studies looking at microbiota−ILC interactions.

## Summary and outlook

The tremendous impact of the microbiota on health and thus the immune system of the host has been appreciated for a long time. The recently discovered field of ILC biology and its importance in the establishment and regulation of the host immune system provides novel insights into the regulation and function of the host−microbial mutualism. In this review, we aimed to give an overview of recent findings on the regulatory interplay between microbiota and ILC function throughout life at steady‐state and during disease. The microbiota is continuously adapting to its environment throughout the lifetime. The synergism of commensals and immune cells has a tremendous impact on the host’s health and immune defense. Just recently, Godinho‐Silva *et al*. deciphered that the circadian clock activator *Antl* is essential for intestinal ILC3 function at steady‐state and upon infection by *C. rodentium*,^161^ revealing that ILC3s are controlled by additional environmental factors. We are just beginning to understand the complexity of these networks, and are excited to decipher this close interaction of microbiota and ILCs during the complete lifespan in the future.

## Disclosures

The authors declare no conflict of interest.
